# Distribution of *Brucella* field strains isolated from livestock, wildlife populations, and humans in Italy from 2007 to 2015

**DOI:** 10.1371/journal.pone.0213689

**Published:** 2019-03-22

**Authors:** Fabrizio De Massis, Katiuscia Zilli, Guido Di Donato, Roberta Nuvoloni, Sandro Pelini, Lorena Sacchini, Nicola D’Alterio, Elisabetta Di Giannatale

**Affiliations:** 1 National and OIE Reference Laboratory for Brucellosis, Istituto Zooprofilattico Sperimentale dell’Abruzzo e del Molise ‘G. Caporale,’ Campo Boario, Teramo, Italy; 2 Dept. of Veterinary Sciences, Univ. of Pisa, Pisa, Italy; Institut National de la Recherche Agronomique, FRANCE

## Abstract

Brucellosis is a major public health problem still prevalent as a neglected endemic zoonosis requiring proactive attention in many communities worldwide. The present study involved analysis of *Brucella* field strains submitted for typing to the Italian National Reference Laboratory for Brucellosis from 2007 to 2015. Strains were identified at the species and biovar levels by classic and molecular techniques according to the World Organisation for Animal Health Manual. In total, 5,784 strains were typed: 3,089 *Brucella abortus* (53.4%), 2,497 *B*. *melitensis* (43.2%), 10 *B*. *ovis* (0.2%), 181 *B*. *suis* (3.1%), and 7 *B*. *ceti* (0.1%). The 2,981 strains from cattle were typed as *B*. *abortus* biovars 1, 3, and 6 (90.1%) and *B*. *melitensis* biovar 3 (9.9%). The 318 strains from water buffalo were typed as *B*. *abortus* biovars 1, 3 (95.9%) and *B*. *melitensis* biovar 3 (4.1%). The 2,279 strains from sheep and goats were typed as *B*. *abortus* biovars 1 and 3 (4.3%); *B*. *melitensis* biovars 1, 3, (95.3%); and *B*. *ovis* (0.4%). The 173 strains from wild boar were typed as *B*. *suis* biovar 2 (98.3%) and *B*. *melitensis* biovar 3 (1.7%). The 11 strains from pigs were typed as *B*. *suis* biovar 2. The 13 strains from humans were typed as *B*. *melitensis* biovar 3. The two strains from horses were typed as *B*. *abortus* biovar 1, while the seven strains from dolphins were typed as *B*. *ceti*. This additional knowledge on the epidemiology of brucellosis in Italy may be useful to formulate policies and strategies for the control and eradication of the disease in animal populations. The animal species affected, biovars typed, geographical origins, and spatial distributions of isolates are herein analyzed and discussed.

## Introduction

Brucellosis is an important zoonotic disease caused by infection with bacteria of the genus *Brucella*. The disease may affect cattle, sheep, goats, pigs, and humans. Having a worldwide distribution, it is one of the most important zoonoses in the Mediterranean and Middle East regions. Eleven species are recognized within the genus [1], each one with individual host preferences, pathogenicity, and epidemiology: *Brucella abortus* (7 biovars), which mainly infects cattle; *B*. *melitensis* (3 biovars), which preferentially infects sheep and goats; *B*. *suis* (5 biovars), which mostly infects pigs; *B*. *canis*, which affects dogs; *B*. *ovis*, which affects sheep; *B*. *neotomae*, which infects the desert wood rat; *B*. *microti*, which affects the common vole [2]; *B*. *ceti*, which infects cetaceans; *B*. *pinnipedialis*, which infects seals [3]; and *B*. *inopinata*, which was isolated from a human breast implant infection [4]. Besides these, *B*. *papionis* and *B*. *vulpis* were recently isolated from the baboon (*Papio* spp.) and red fox (*Vulpes vulpes*), respectively [5, 6]. In the Mediterranean area, bovine brucellosis is typically caused by *B*. *abortus* while ovine and caprine brucellosis are mainly caused by *B*. *melitensis*, although cross-species infections may occur [7]. The typical clinical sign of the infection in affected animals is the occurrence of abortion (although this depends on whether the infection is recent or has been chronically present) as well as low fertility and milk production. However, the disease can be present in an animal for several years without clinical signs [8]. While the disease incidence and prevalence may vary widely among countries, brucellosis caused by *B*. *melitensis* is by far the most important clinically apparent disease in humans [9]. Human brucellosis is a systemic infectious disease with varying clinical manifestations. Patients often develop fever of unknown origin with an insidious clinical onset. The disease is often difficult to diagnose because of its similarities with other febrile diseases, such as malaria or other undulating fevers, and it occurs as a subacute or chronic illness that is generally not lethal [10, 11]. The acute stage is characterized by nonspecific symptoms similar to a flu-like or septicemic illness. Clinical manifestations may be the effect of many disorders such as osteoarticular, dermal, gastrointestinal, respiratory, cardiovascular, and neurologic involvement, thus mimicking many other infectious and noninfectious diseases. Direct invasion of the central nervous system may occur in about 5% of cases (*B*. *melitensis*), and meningitis or meningoencephalitis is the most common finding. *Brucella* spp. meningitis can be acute or chronic. It often occurs late in the disease course; however, it may also be the presenting manifestation [12]. However, although their occurrence is rare, endocarditis and neurobrucellosis may be fatal.

Human brucellosis is one of the most common bacterial zoonotic infections worldwide, but it remains an often regionally neglected disease. Currently, *B*. *melitensis*, *B*. *abortus*, and *B*. *suis* have a major impact on public health. Infection in humans may occur by ingestion of contaminated dairy products (especially raw milk in developing countries) and in occupationally exposed groups. A few cases of human brucellosis caused by *B*. *canis* have also been described, while *B*. *ovis* infection has not been unequivocally associated with human disease. No cases of infection with *B*. *neotomae* have been recorded; even if this species is confirmed to be a human pathogen, infection would be unlikely given the rarity and restricted geographic distribution of this organism. Besides the “classic” species, some recently discovered “new” *Brucella* species have demonstrated their zoonotic potential, such as *B*. *ceti* [13, 14, 15, 16, 17, 18, 19, 20, 21, 22, 23]. In the European Union, 619 cases of human brucellosis were reported in 2008, and this figure decreased to 437 cases in 2015. The highest incidence was recorded in some member states still not officially free from bovine and sheep and goat brucellosis (Italy, Portugal, Greece, and Spain).

The geographical distribution of animal brucellosis is constantly changing. As new foci emerge in infected areas or re-emerge in previously free areas, new cases of animal (and consequently human) brucellosis may emerge or re-emerge. Therefore, a sound knowledge of the epidemiology of the disease in animals, particularly with respect to the geographical characterization of the species and biovars of *Brucella*, is of utmost importance to establish and implement reliable and efficient control measures against brucellosis in a “One Health” perspective. Knowledge of the prevailing species and biovars of *Brucella* field strains isolated in animal outbreaks is therefore an important epidemiological tool to support the classic epidemiological investigation techniques. Characterization of the isolates linked with the epidemiologic data may help to identify the correlation between cases of the disease in animals and humans within a cluster or outbreak. This is essential to formulate policies and strategies for the control of brucellosis in animal populations and to trace back the introduction of new strains, thus helping to avoid the spread of brucellosis in humans.

The aim of this paper is to provide an overview of the *Brucella* strains isolated from livestock, wild animal species, and humans in Italy from 2007 to 2015. From a “One Health” perspective, the identification of isolated species and biovars of *Brucella* field strains is essential to fully understand the epidemiology of the disease and to trace back the sources of infection, thereby improving the prevention of infection in humans and the outcome of brucellosis eradication programs in animals.

## Materials and methods

### Bacterial isolates

In total, 5,784 *Brucella* isolates from confirmed cases of animal and human brucellosis in Italy from 2007 to 2015 were included in the study. Samples were collected from organs and tissues of livestock slaughtered in the framework of the national Brucellosis Eradication Plan or wild animals found dead and submitted for necropsy by competent authorities. Animal welfare during slaughtering procedures was ensured by veterinary services as required by legislation [24].

All strains were isolated by the local Italian Istituti Zooprofilattici Sperimentali (State Veterinary Laboratories) at *Brucella* spp. level and then sent to the Istituto Zooprofilattico Sperimentale dell’Abruzzo e del Molise ‘G. Caporale,’ Teramo, Italy [National and World Organisation for Animal Health Manual (OIE) Reference Centre for Brucellosis] for species and biovar typing, according to the rules stated in the Ministerial Order of 14 November 2006. Specimens were transported and delivered in accordance with the World Health Organization (WHO) safety guidelines [25] and the IATA—Infectious Substances Shipping Guidelines—WHO–“Guidance on Regulations for the Transport of Infectious Substances 2015–2016” [26, 27]. The collection of data such as animal species, region of origin, and geographic coordinates was standardized using a form available on the National Brucellosis Reference Centre website (www.izs.it). The *Brucella* polyvalent and monospecific *Brucella* A and M antisera were supplied by the Food and Agriculture Organization/WHO Collaborating Centre for Research on Brucellosis (Veterinary Laboratories Agency, Weybridge, UK). All *Brucella* field isolates were subcultured in *Brucella* agar base and stored using the Microbank system (Pro-Lab Diagnostics, Toronto, Ontario, Canada) at −80°C.

### Isolation procedures

According to the technique described in the OIE Manual of Diagnostic Tests and Vaccines [28, 29], the primary isolation of *Brucella* was performed by culturing the samples in *Brucella* broth supplemented with Farrell’s mix of antibiotics [30] and on *Brucella* agar (Oxoid, Basingstoke, Hampshire, UK) supplemented with 5% horse serum and antibiotics at the following amounts per 1 L of media: bacitracin (25 000 IU), polymyxin B (5000 IU), natamycin (50 mg), nalidixic acid (5 mg), nystatin (100 000 IU), and vancomycin (20 mg). The broth was incubated at 37°C ± 2°C in an atmosphere supplemented with 5% to 10% CO_2_ (v/v) for up to 6 weeks. From the broth, two plates per sample were inoculated each week: one plate was incubated in aerobiotic conditions at 37°C ± 2°C and the other in an atmosphere supplemented with 5 to 10% CO_2_ (v/v) at 37°C ± 2°C. The plates were observed after 3 days and then daily to identify *Brucella*-like colonies. The plates were discarded if no specific growth was evident after 7 to 10 days of incubation. Suspected colonies were subcultured onto serum dextrose agar from which subsequent growth was examined microscopically using Gram stain and biochemical (urease, oxidase, and catalase) and motility tests.

### Identification methods

Identification were performed with AMOS (*abortus*, *melitensis*, *ovis*, *suis*) polymerase chain reaction (PCR) (i.e., AMOS-PCR) and PCR-restriction fragment length polymorphism techniques (PCR-RFLP). AMOS-PCR is a multiplex PCR designed to detect four species of *Brucella* [31]. The PCR Master Mix by Promega (Madison, WI, USA) was used. The assay exploits the polymorphism arising from species-specific localization of the insertion sequence IS711 in the *Brucella* chromosome. Individual biovars within a species are not differentiated [32]. Amplification was performed for 33 cycles in a thermal cycler (GeneAmp PCR System 9700; PE Applied Biosystems, Waltham, MA, USA) at an annealing temperature of 60°C. Amplicons were checked by fluorescence after electrophoresis in a 1% agarose gel with ethidium bromide.

**Fig 1:**
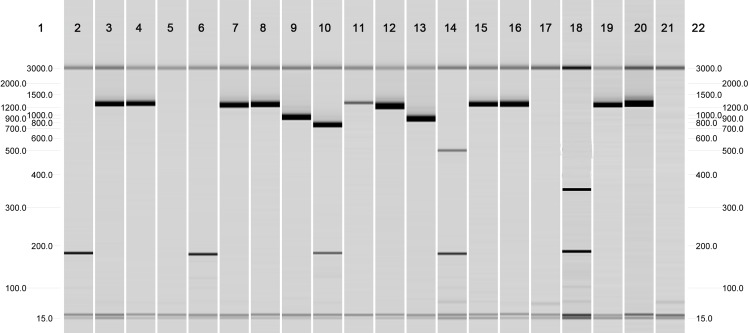
Brucella AMOS-PCR profiles: In order to identify *Brucella* species and biovars, for each strain, 4 PCR have been performed: AMOS multiplex PCR, Omp 2a PCR, Omp 2b PCR and Omp 31 PCR. In this image, this four PCR have been grouped to obtain a good glance and easily identify the *Brucella* profile. *Lane 1* and 22 Marker molecular weights; *Lane* 2–5: Amos (2)-Omp2a (3)-Omp2b (4)-Omp31 (5) *B*. *abortus* biovar 3,5,6,9; *Lane* 6–9: Amos (6)-Omp2a (7)-Omp2b (8)-Omp31 (9) *B*. *suis* biovar 2,3,4,5; *Lane* 10–13: Amos (10)-Omp2a (11)-Omp2b (12)-Omp31 (13) *B*. *melitensis* biovar 1,2,3; *Lane* 14–17: Amos (14)-Omp2a (15)-Omp2b (16)-Omp31 (17) *B*. *abortus* biovar 1,2,4; *Lane* 18–21: Amos (18)-Omp2a (19)-Omp2b (20)-Omp31 (21) *B*. *ovis*.

Three different PCRs were used to amplify three outer membrane protein genes of *Brucella*: *omp2a*, *omp2b*, and *omp31* (**Fig 1)**. The amplicons of the *omp2a*, *omp2b*, and *omp31* genes were digested by endonucleases (Pst I, Hinf I, Taq I, Ava II, and Nco I), and the products of digestion were checked by fluorescence after electrophoresis in 3% agarose gel in the presence of ethidium bromide. The specific biovar pattern was obtained by crossing the results of the single *omp* restrictions [33, 34, 35, 36].

For B.abortus and B.suis, to complete the biovars differentiation, addictional tests were performed agglutination with anti-A, anti-M, and anti-R monospecific sera; the production of H_2_S; CO_2_-dependence; and growth in the presence of basic fuchsin and thionin at a final concentration of 20 μg/ml.

## Results

The total number of strains examined is shown in **Table 1**. Overall, 5,784 strains submitted from 13 regions in Italy were analyzed. Strains isolated from livestock were obtained from cattle (*Bos taurus*, 2,981 isolates), water buffalo (*Bubalus bubalis*, 318 isolates), sheep (*Ovis aries*, 1,849 isolates), goats (*Capra hircus*, 430 isolates), pigs (*Sus scrofa domesticus*, 11 isolates), wild boar (*Sus scrofa ferus*, 173 isolates), horses (*Equus caballus*, 2 isolates), and dolphins (*Stenella coeruleoalba*, 7 isolates). Thirteen strains included in the study were isolated from humans. The strains isolated from cattle were *B*. *abortus* biovars 1, 3, and 6. *Brucella melitensis* biovar 3 was also isolated from cattle in several regions. In water buffalo, *B*. *abortus* biovars 1 and 3, and *B*. *melitensis* biovar 3 were identified. The strains isolated from sheep and goats were mainly *B*. *melitensis* biovar 3. *B*. *melitensis* biovar 1, and *B*. *abortus* biovar 1 and 3 were also isolated from these animal species. The only strain isolated from pigs was *B*. *suis* biovar 2. The same biovar was also isolated from wild boars. *Brucella abortus* biovar 1 was isolated from horses. *Brucella ceti* was isolated from some specimens of *Stenella coeruleoalba* found dead on the Italian coast. Infection was detected in 13 humans and was caused by *B*. *melitensis* biovar 3 in all cases. The relative percentage of isolation within animal species is shown in **Table 2.** The strains identified from cattle showed a high prevalence of *B*. *abortus* biovar 3 isolates (84.5%) followed by *B*. *melitensis* biovar 3 (9.9%) and *B*. *abortus* biovars 1 and 6 (5.5% and 0.1%, respectively) (**Table 2)**. In water buffalo, most isolates were *B*. *abortus* biovars 1 and 3 (48.7% and 47.2%, respectively) (**Table 2)**. Some isolates of *B*. *melitensis* biovar 3 (4.1%) were also identified (**Table 2)**. The strains isolated from sheep showed a high prevalence of *B*. *melitensis* biovar 3 (95%) (**Table 2)**. A small percentage of isolates were *B*. *abortus* biovars 1 and 3 (0.1% and 4.3%, respectively) and *B*.*ovis* (0.5%) (**Table 2)**. The isolates from goats were *B*. *melitensis* biovar 3 (96.3%) and *B*. *abortus* biovar 3 (3.5%) (**Table 2)**. A small percentage of *B*. *melitensis* biovar 1 (0.2%) was identified. A total of 98.3% of isolates from wild boar were typed as *B*. *suis* biovar 2 (**Table 2)**, while 1.7% of *B*. *melitensis* biovar 3 was isolated (**Table 2)**. All isolates from pigs were *B*. *suis* biovar 2, while all isolates from horses were *B*. *abortus* biovar 1. All isolates from dolphins were *B*. *ceti*, and all isolates from humans were *B*. *melitensis* biovar 3 (**Table 2)**.

**Table 1:** Total numbers of *B*. *abortus*, *B*. *melitensis*, *B*. *ovis*, *B*. *suis*, *and B*. *ceti* biovars isolated from 2007 to 2015.

	*B*. *abortus*			*B*. *melitensis*			*B*. *ovis*	*B*. *suis*	*B*. *ceti*	Total
	1	3	6	1	2	3	-	2	-	
**Cattle** **(*Bos taurus*)**	164	2,518	3	-	-	296	-	-	-	**2,981**
**Water buffalo** **(*Bubalus bubalis*)**	155	150	-	-	-	13	-	-	-	**318**
**Sheep** **(*Ovis aries*)**	2	80	-	-	-	1,757	10	-	-	**1,849**
**Goats** **(*Capra hircus*)**	-	15	-	1	-	414	-	-	-	**430**
**Wild boar** **(*Sus scrofa ferus*)**		-	-	-	-	3	-	170	-	**173**
**Pigs** **(*Sus scrofa domesticus*)**	-	-	-	-	-	-	-	11	-	**11**
**Horses** **(Equus caballus)**	2	-	-	-	-	-	-	-	-	**2**
**Dolphins** **(*Stenella coeruleoalba*)**	-	-	-	-	-	-	-	-	7	**7**
**Humans** **(*Homo sapiens sapiens*)**	-	-	-	-	-	13	-	-	-	**13**
**Total**	**323**	**2,763**	**3**	**1**	**0**	**2,496**	**10**	**181**	**7**	**5,784**

**Table 2:** Percentages of *B*. *abortus*, *B*. *melitensis*, *B*. *ovis*, *B*. *suis*, and *B*. *ceti* biovars isolated from 2007 to 2015.

	*B*. *abortus*			*B*. *melitensis*			*B*. *ovis*	*B*. *suis*	*B*. *ceti*	Total
	1	3	6	1	2	3	-	2	-	
**Cattle** **(*Bos taurus*)**	5.5%	84.5%	0.1%	-	-	9.9%	-	-	-	
**Water buffalo** **(*Bubalus bubalis*)**	48.7%	47.2%	-	-	-	4.1%	-	-	-	
**Sheep** **(*Ovis aries*)**	0.1%	4.3%	-	-	-	95%	0.5%	-	-	
**Goats** **(*Capra hircus*)**	-	3.5%	-	0.2%	-	96.3%	-	-	-	
**Wild boar** **(*Sus scrofa*)**	-	-	-	-	-	1.7%	-	98.3%	-	
**Pigs** **(*Sus scrofa*)**	-	-	-	-	-	-	-	100%	-	
**Horses** **(*Equus caballus*)**	100%	-	-	-	-	-	-	-	-	
**Dolphins** **(*Stenella coeruleoalba*)**	-	-	-	-	-	-	-	-	100%	
**Humans** **(*Homo sapiens sapiens*)**	-	-	-	-	-	100%	-	-	-	
**Total**	**5.5%**	**47.8%**	**0.1%**	**0%**	**0%**	**43.2%**	**0.2%**	**3.1%**	**0.1%**	**100%**

**Fig 2:**
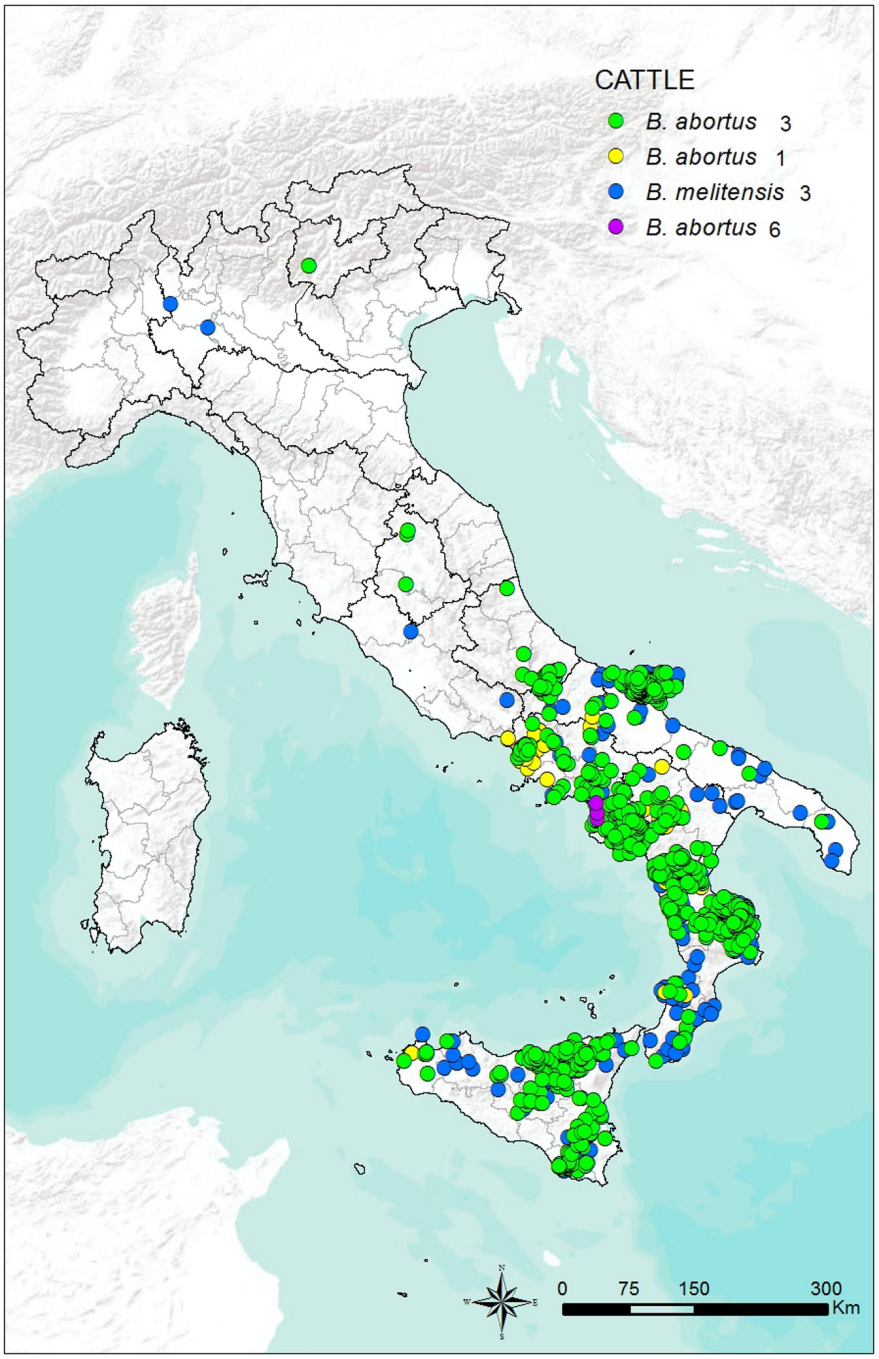
Geographical distribution of the 2,981 *Brucella* strains isolated from cattle in Italy from 2007 to 2015.

**Fig 3:**
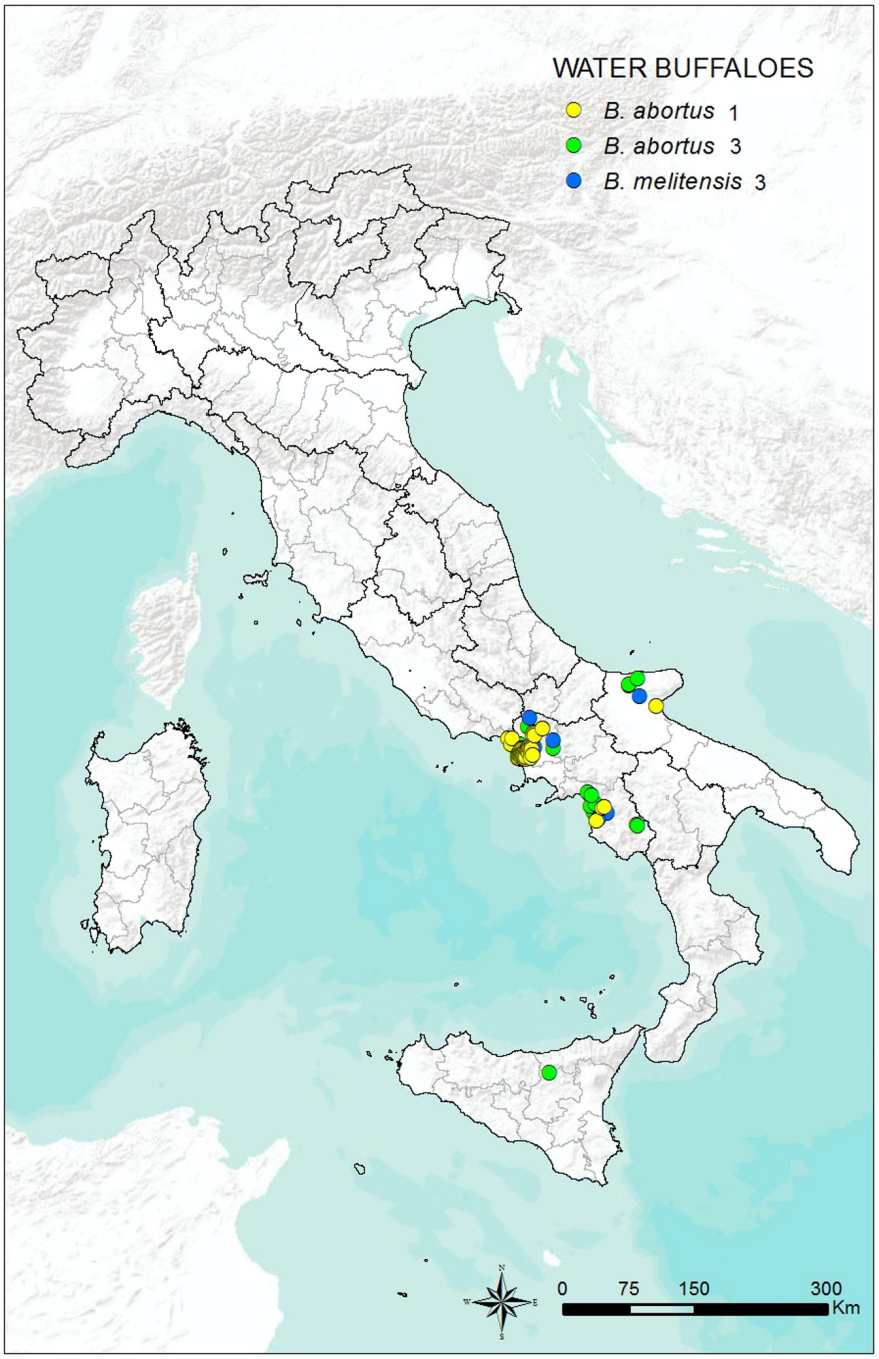
Geographical distribution of the 322 *Brucella* strains isolated from water buffalo in Italy from 2007 to 2015.

The geographical distribution of the 2,981 *Brucella* strains isolated from cattle is shown in **Fig 2**, while the geographical distribution of the 318 *Brucella* strains isolated from water buffalo is shown in **Fig 3**.

**Fig 4:**
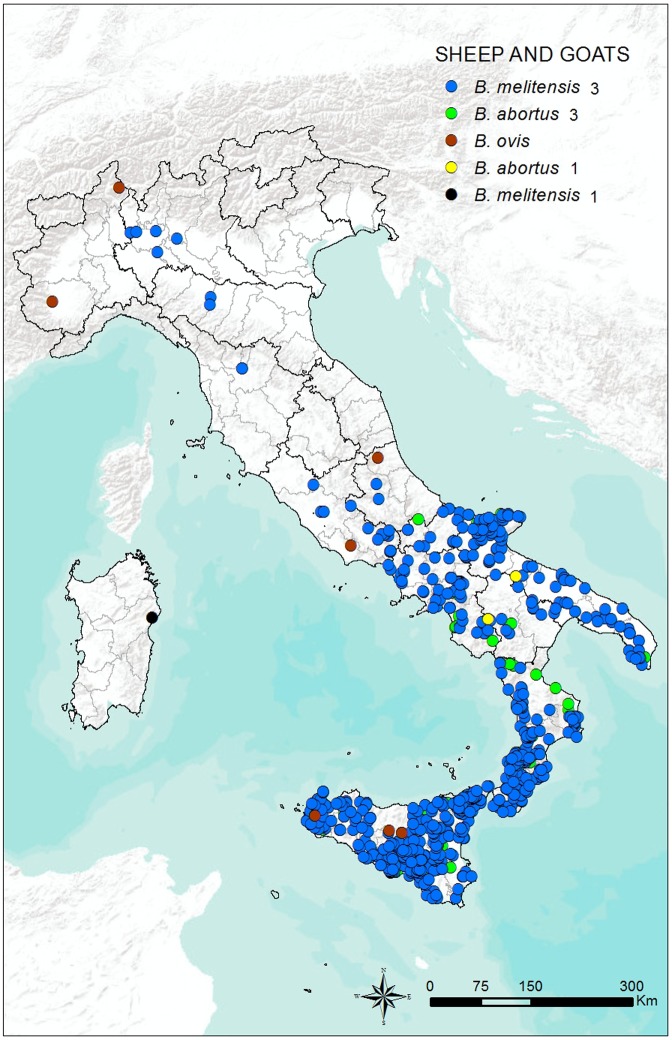
Geographical distribution of the 2,342 *Brucella* strains isolated from sheep and goats in Italy from 2007 to 2015.

The geographical distribution of the 2,279 *Brucella* strains isolated from sheep and goats is shown in **Fig 4.** *Brucella melitensis* biovar 3 represented 95.3% of the total number of strains isolated in sheep and goats in Italy (**Table 2)**.

**Fig 5:**
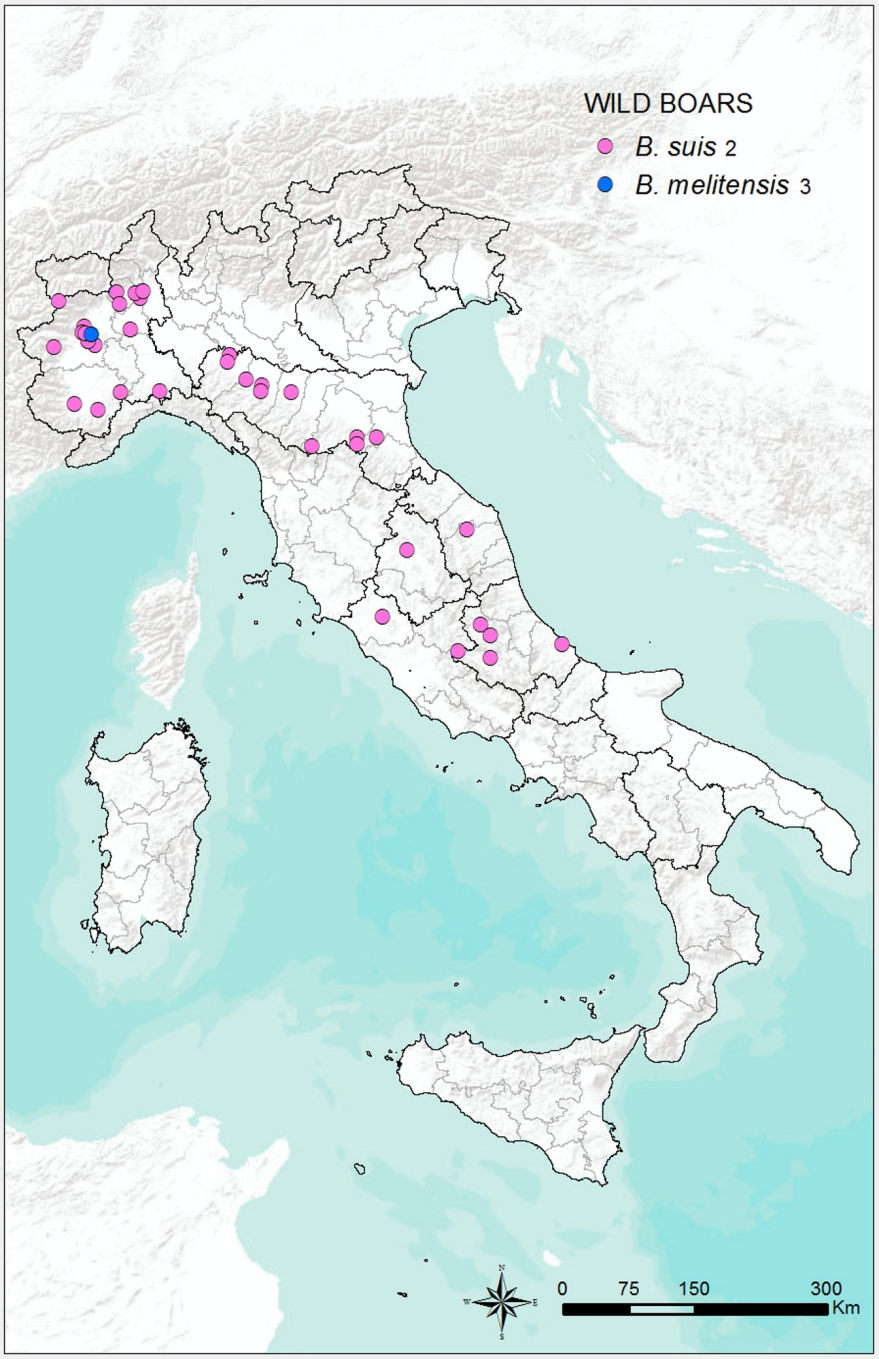
Geographical distribution of the 173 *Brucella* strains isolated from wild boars in Italy from 2007 to 2015.

**Fig 6:**
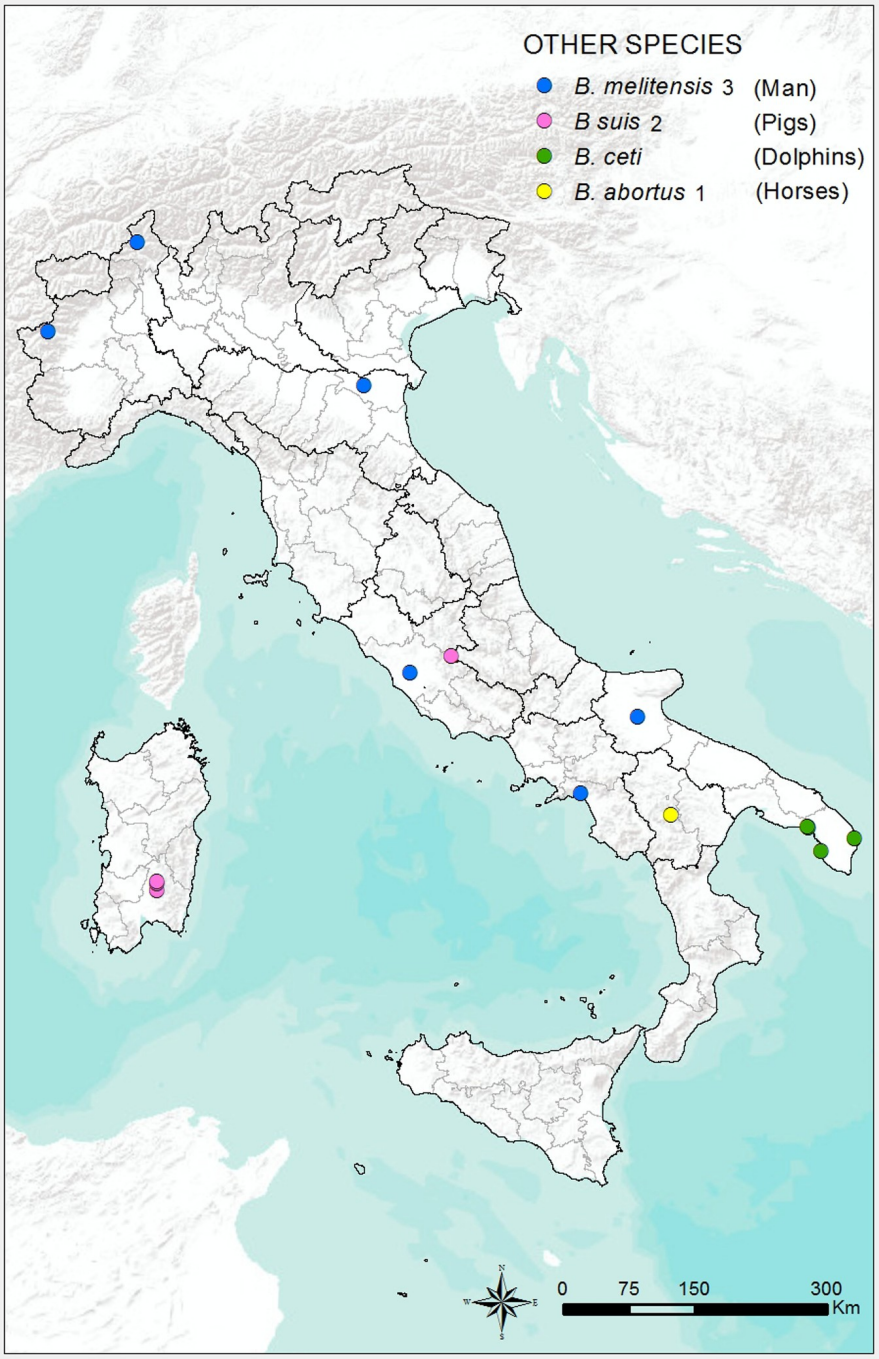
Geographical distribution of the 33 *Brucella* strains isolated from humans, pigs, dolphins, and horses in Italy from 2007 to 2015.

The geographical distribution of the 173 *Brucella* strains isolated from wild boars is shown in **Fig 5**, while the distributions of the 11 *Brucella* strains isolated from pigs, the 2 strains isolated form horses, the 7 strains isolated from dolphins, and the 13 strains isolated from humans are shown in **Fig 6**.

## Discussion

The additional knowledge provided by this study on the identification and epidemiology of the prevailing species and biovars of *Brucella* that affect livestock and humans in Italy may be crucial for formulating policies and strategies for the control of brucellosis in animal populations, thus protecting human health. Although the resolution level provided by the identification of the Brucella isolated at species and biovar level may be lower than the one provided by modern molecular methods, or interesting new approach [37, 38], instead they remain methods with a discriminatory power well described and accepted in the international scientific community as well as in the international guidelines for trade in animal health (OIE, 2017).

The purpose of species and biovar identification is different from the determination of the genetic diversity of the strains isolated in the animal species or from a phylogenetic analysis of the isolates; it represents instead a description of the strains circulating in given territories in relation to a given space location and a given time period. In other papers the authors have discussed and evaluated different aspects of genetic approaches to describe the epidemiological situation from a molecular point of view or phylogenetic [39, 40, 41, 42, 43, 44, 45] point of view. However, the current classification of Brucella in species and biovar has been the outcome of epidemiological analysis over time rather than phylogenetic molecular analysis therefore is still today susceptible to better describe the distribution of Brucella in the field in Italy even in the light of the length of the period considered and the number of strains analyzed.

### Cattle and water buffalo

Previous studies have isolated of six of the eight known *B*. *abortus* biovars in Italy, namely biovars 1, 2, 3, 4, 6, and 7 [46]. However, from 2007 to 2015, only biovars 1, 3, and 6 were isolated. The absence of isolation of biovars 2, 4, and 7 may suggest that these biovars have been eradicated from the country and that these strains may currently be considered exotic. In line with the distribution of the water buffalo population in Italy, most strains were isolated in Campania, the Italian region where 74.2% of the national stock of this species is farmed (**Table 2)** (286,946 of 386,731 total heads in Italy as of 31 December 2016) (http://statistiche.izs.it/portal/page?_pageid=73,12918&_dad=portal&_schema=PORTAL, accessed on 31 January 2017). Several *B*. *melitensis* isolates were also recorded in the present study, both in cattle and water buffalo (**Figs 1 and 2)**. The percentage of *B*. *melitensis* isolates among the total number of strains submitted for typing was 9.9% in cattle and 4.1% in water buffalo (**Table 2)**. The number of isolates was not very high, and it dropped significantly compared with previous years. However, *B*. *melitensis* can be shed in milk by infected cows, thus constituting a potential hazard for milk and milk product consumers. Infection among farm workers, butchers, and veterinarians may also occur as an occupational disease while handling infected animals or organs after slaughter [47]. Moreover, the occurrence of *B*. *melitensis* infection in cattle is of particular concern, given that *B*. *abortus* vaccines do not effectively protect against *B*. *melitensis* infection. In the past, cattle were the major source of human infection in most countries, and programs to eradicate the disease have been aimed largely at bovine brucellosis. Success has been achieved in northern and eastern European countries, Australia and New Zealand, Japan, Canada, and the US. Cattle are also the source of human brucellosis in most African countries, where large numbers of cattle are maintained and drinking raw milk is a custom. In countries with near universal pasteurization of milk, brucellosis has become an occupational disease and it remains a serious zoonosis for general population in the areas of the world where *B*. *melitensis* is endemic in sheep and goats [48].

### Sheep and goats

*Brucella abortus* strains were also isolated in these species, although seldom reported in the past, and the possibility of shedding this strain in milk has been documented at least for sheep [49]. Nevertheless, risk factors including husbandry practices and exposure potential should be evaluated to determine the need to test sheep and goats that may have been exposed to cattle infected with *B*. *abortus*. The same risk factor evaluation should be applied to cattle exposed to sheep and goats infected with *B*. *melitensis*. *Brucella ovis* was isolated in sheep in the Piedmont, Abruzzi, Sicily, and Lazio regions (**Fig 4)**. This suggests a widespread presence of the infection in the Italian sheep population; however, the distribution of this strain in sheep populations should be more thoroughly assessed. Actually, reliable information about the distribution of this strain is scarce; this strain has never been actively investigated because is not considered pathogenic for humans. Previous studies in Italy regarding the impact of animal brucellosis on humans have suggested an overlap between the distribution of disease in humans and that in cattle and ovicaprine populations. However, from 1970 to date, *B*. *melitensis* has been the pathogen isolated most frequently in human cases, accounting for more than 99% of *Brucella* spp. isolated from humans. Therefore, the brucellosis problem in Italy seems to be focused more on infection in the ovicaprine than cattle population.

### Wild boars and other species

*Brucella suis* biovar 2 is the main strain responsible for brucellosis in wild boars in Europe [50]. This was confirmed by the results of the present study, in which *B*. *suis* biovar 2 was the main strain isolated; this is also in agreement with previous Italians records [51, 52]. However, a small percentage (1.7%) of *B*. *melitensis* biovar 3 was isolated from wild boar samples (**Table 2)** collected during monitoring activities carried out in a regional Park of Piedmont called “La Mandria,” a natural reserve from the 16th century that was used as a hunting reserve of the Savoia court. The occurrence of *B*. *melitensis* infection in wild boar in that area might have been a consequence of transmission between the wild boar population and wild ruminants, allowed by strict contact in a closed environment. *Brucella suis* biovar 2 was the only strain isolated from pigs, as has been reported in the past [53]. However, the presence of this strain in Italian pig farms may be largely underestimated because no specific surveillance plan has been implemented in the country. Most human infections derived from swine are caused by *B*. *suis* biovars 1 and 3. These biovars are most prevalent in Latin America, Southern Asia, China, and Oceania. *Brucella suis* biovar 2 is largely restricted to continental Europe and is maintained in the wild hare populations in an area extending eastward from the Atlantic Coast to the Ural Mountains and southward from the shores of the Baltic Sea to the Mediterranean. The agent is transmitted sporadically to domesticated pigs and these, together with infected wild boars and hares, are a potential source of human infection. However, this biovar seems to possess a low virulence for man, and few verified cases of human brucellosis caused by it have been recorded.

A strain of *B*. *abortus* biovar 1 was isolated in a horse located in the Basilicata region; this biovar was cultured by pathologic material collected from the supraspinous bursa. However, other species are farmed in the same environment, particularly cattle, the natural host of *B*. *abortus* biovar 1. Sporadic cases of horses infected with *B*. *abortus* have been reported. In some cases, infection may remain asymptomatic, but in other cases the infection may be associated with a variety of clinical manifestations, including osteoarthritis and osteomyelitis, abortion, and infertility. Naturally acquired *B*. *abortus* infection in horses commonly manifests as chronic bursal enlargement of the neck and withers or navicular bursa, referred to as fistulous withers or poll evil, respectively [54, 55, 56, 57, 58, 59]. Even if it is likely that abortions in mares due to *B*. *abortus* may pose a risk for transmission to cattle (and therefore a risk of human infection), documentation of this occurrence is lacking [60].

Infection by *B*. *ceti* is common in cetaceans, but only small proportions of infected cetaceans display pathological signs associated with brucellosis, excluding the striped dolphin (*Stenella coeruleoalba*). This may suggest that many infected cetaceans overcome infection, perhaps remaining as carriers and potential *Brucella* shedders [61]. The record of seven isolates of *B*. *ceti* confirms the presence of the bacterium in the Tyrrhenian and Adriatic Seas. Although *Brucella* strains from land animals have not been identified in cetaceans, *B*. *ceti* strains have been isolated from human cases, stressing the zoonotic potential of this strain. However, despite the few human cases attributed to *Brucella* isolated from marine mammals, the magnitude of the risk that *B*. *ceti* represents for humans remains unknown.

All isolates from human samples were *B*. *melitensis* biovar 3, and all were identified in hospitals from the Lombardia, Piedmont, Campania, Emilia Romagna, Lazio, and Puglia regions. This is consistent with previous findings where, in Italy, brucellosis caused by *B*. *melitensis* was the most important clinically apparent disease recorded in humans. However, all human cases reported in the present study were notified in hospitals. Actually, because the Italian legislation requires human cases of brucellosis to be reported by the hospitals at which the cases are investigated, the site of disease notification is often different from the site at which the patient acquired the infection. In our cases, strains were received by some of hospitals for supporting the isolation or the typing process, and this may also explain the lower rate of isolation in humans than in animals.

## Supporting information

S1 File Supporting information. (XLSX)
